# Successful transurethral endoscopic removal of a toothpick embedded in the bladder wall: a rare Case Report

**DOI:** 10.3389/fsurg.2025.1648760

**Published:** 2025-12-01

**Authors:** Tao Ma, Jianjun Guo, Guihua Cao, Qian Cai, Yin Yu, Lijun Zhou

**Affiliations:** 1 Department of Urology, The People’s Hospital of Leshan, Leshan, China; 2 Department of Urology, Affiliated Hospital of Yunnan University (Second People’s Hospital of Yunnan Province, Ophthalmic Hospital of Yunnan Province), Kunming, Yunnan, China

**Keywords:** bladder foreign body, ultrasound-guided transurethral surgery, wooden toothpick ingestion, unexplained abdominal pain, CT three-dimensional imaging

## Abstract

**Background:**

This report describes a rare case of a wooden toothpick embedded in the bladder muscular layer and shares the experience of successful diagnosis and treatment.

**Case presentation:**

A 29-year-old female patient with a history of cesarean section was admitted due to persistent lower abdominal pain and dysuria lasting one month. Preoperative three-dimensional CT imaging was performed, using a crucian carp with an inserted injection needle as a density reference. Density comparisons between the foreign body, needle, and fishbone suggested that the foreign body was unlikely a retained needle or fishbone. Considering the patient's dietary habits, it was suspected that the object was an accidentally swallowed wooden toothpick. The patient underwent ultrasound-guided transurethral surgery, during which a wooden toothpick approximately 3.4 cm long, 4 mm wide at its midsection, with a rough surface, was successfully removed. Postoperative imaging confirmed the complete removal of the foreign body, and the patient recovered well. During a six-month follow-up, the patient reported no urinary or abdominal symptoms.

**Conclusion:**

A detailed patient history and imaging studies are crucial for diagnosing unexplained bladder foreign bodies. Ultrasound-guided transurethral cystoscopic surgery is an effective method for removing bladder foreign bodies. This case provides valuable insights for managing and treating similar complex cases.

## 1 Introduction

Bladder foreign bodies can be classified based on their entry route as follows: iatrogenic retention, transurethral insertion (intentional or accidental), and migration from other internal organs or tissues (1). Approximately 80%–90% of gastrointestinal foreign bodies pass spontaneously within one week, with only a small proportion (<1%) causing complications such as gastrointestinal perforation (2, 3). Wooden toothpicks account for 9% of reported ingested foreign bodies, but complications involving toothpicks are rare (<0.1%) (4, 5). In most reported cases, gastrointestinal foreign bodies remain within the peritoneal cavity without penetrating adjacent organs (6). Migration of foreign bodies from the gastrointestinal tract to the urinary system is uncommon, and migration specifically to the bladder wall is even rarer (7–11). The migration pathway often remains unclear.

**Figure 1:**
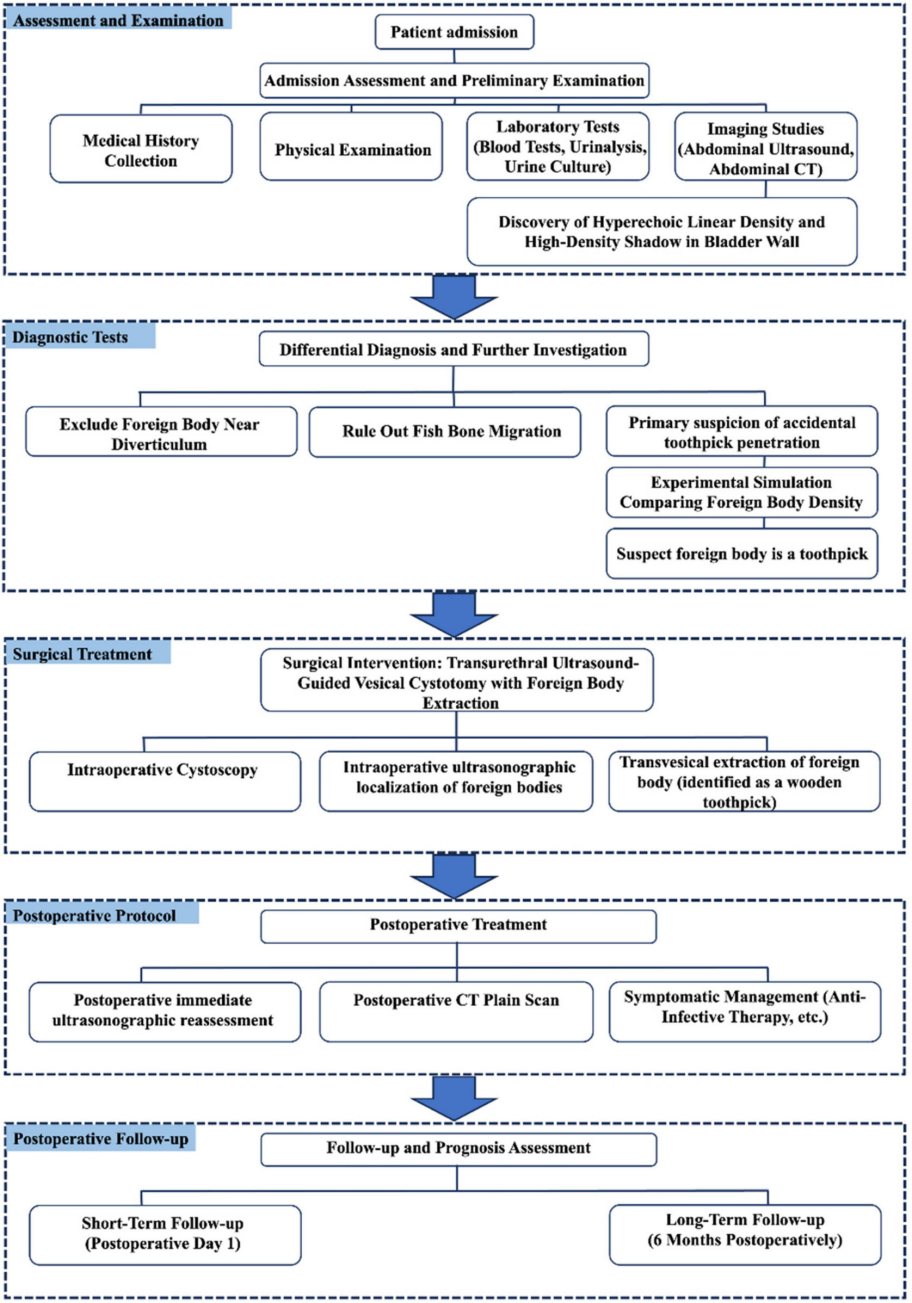
This flowchart illustrates the medical diagnosis and treatment protocol for foreign body extraction. The process commences with patient admission, evaluation, and preliminary examination, followed by diagnostic workup to rule out other foreign bodies and suspect accidental toothpick penetration. For surgical management, transurethral bladder foreign body extraction under ultrasound guidance is selected. Postoperative reassessment includes ultrasound reexamination, computed tomography (CT) scan, and symptomatic supportive care, with postoperative follow-up divided into short-term and long-term phases..

In this report, we present a unique case involving a foreign body embedded in the bladder wall. Preoperative computed tomography (CT) three-dimensional imaging was performed using a syringe needle inserted into a crucian carp for radiodensity comparison. This method allowed preliminary exclusion of a fishbone or needle. Considering the patient's dietary and lifestyle habits and relevant literature (11–13), we hypothesized that the foreign body was a wooden toothpick accidentally swallowed and migrated to the bladder via gastrointestinal perforation. The final diagnosis was confirmed upon endoscopic removal, identifying the foreign body as a wooden toothpick (Figure 1).

## 2 Case report

**Figure 2:**
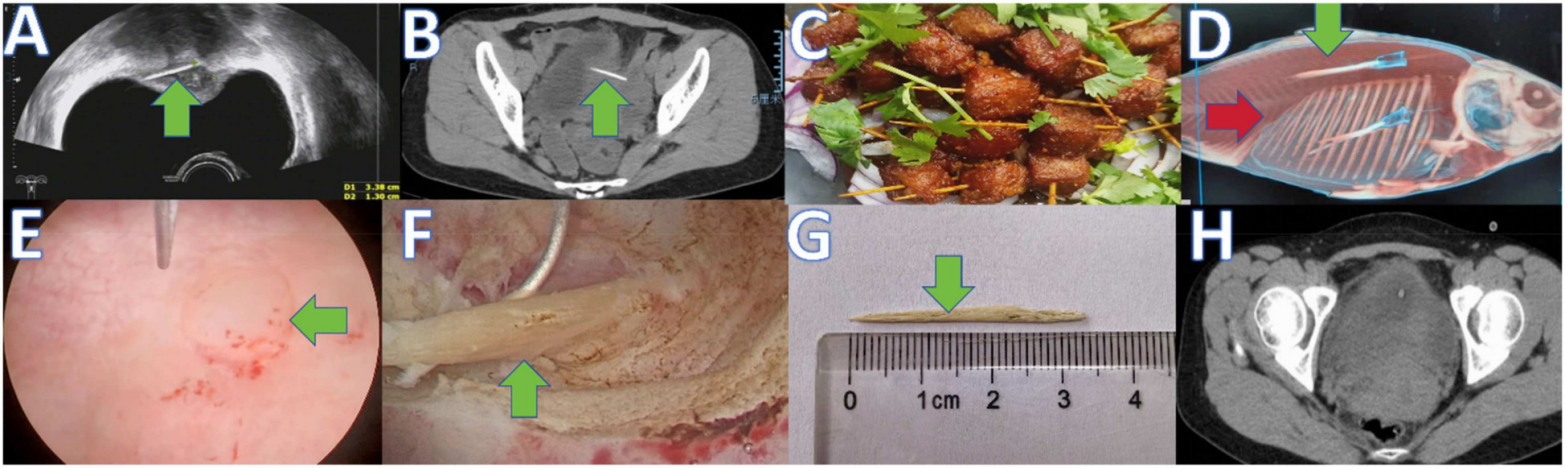
A series of eight images labeled A through H. **(A)** Preoperative abdominal ultrasound showing a foreign body in the bladder muscular layer (green arrow). **(B)** Preoperative CT showing a foreign body in the bladder muscular layer (green arrow). **(C)** Traditional Sichuan dish (wooden-toothpick beef). **(D)** Three-dimensional CT of crucian carp: fishbone (red arrow) and 5 mL syringe needle (green arrow). **(E)** Bulging at the foreign body site on the posterior bladder wall before resection (green arrow). **(F)** Intraoperative transurethral removal of the bladder foreign body (green arrow). **(G)** Removed specimen (wooden toothpick). **(H)** Postoperative CT showing no abnormality after removal.

The patient was a 29-year-old female who presented with a one-month history of lower abdominal pain and dysuria. Approximately one month earlier, she developed mild, intermittent lower abdominal pain without clear precipitating factors. The pain gradually intensified but remained tolerable. There was no visible gross hematuria, flank pain, or fever. The patient did not seek medical care and received no treatment. Physical examination: Vital signs were stable. No tenderness or percussion pain was found in the renal regions, along the ureteral course, or over the bladder. Murphy's sign was negative. The patient had undergone a cesarean section at our hospital two years prior. Laboratory tests: Mild leukocytosis was present in peripheral blood (10.14 × 10^9^/L; reference 4–10 × 10^9^/L). Urinalysis showed no pyuria, and urine culture was negative. Imaging: Abdominal ultrasound demonstrated a linear hyperechoic lesion within the bladder muscular layer (measured 3.3 cm × 0.3 cm on Figure 2A). Abdominal CT revealed a strip-like high-density image in the muscular layer of the posterior bladder wall (measured 3.4 cm × 0.4 cm on Figure 2B). On further questioning, the patient reported a preference for wooden-toothpick beef (Figure 2C) and crucian carp. She regularly used wooden toothpicks to clean her teeth before bed. She denied self-insertion of objects into the urethra and was uncertain whether she had accidentally swallowed a toothpick or fishbone. She suspected that a needle might have been retained during the prior cesarean section at our hospital.

To clarify the diagnosis, an experimental simulation was performed with institutional approval (Leshan People's Hospital). A 250 g crucian carp was purchased and two 5 mL syringe needles (commonly used to inject oxytocin during cesarean section) were implanted into the fish body. The fish underwent three-dimensional CT imaging (Figure 2D). The radiodensity of the patient's foreign body was found to lie between that of a fishbone and a needle (Hounsfield units), preliminarily excluding iatrogenic retention of a syringe needle and migration of a fishbone. Given the patient's dietary habits, the foreign object was considered most likely a wooden toothpick.

The patient underwent ultrasound-guided transurethral foreign body removal under combined intravenous and inhalational general anesthesia. The operation lasted approximately 1.5 h with estimated blood loss of ∼15 mL. The main surgical goals were precise localization of the foreign body and avoidance of bladder perforation. During cystoscopy with an F26 resectoscope, a bulging on the posterior bladder wall was observed. No obvious mucosal discoloration or tumor-like lesion was seen (Figure 2E). Ultrasound-guided resectoscope examination identified a linear hyperechoic lesion within the muscular layer, oriented transversely and not communicating with the bladder lumen. Under ultrasound guidance, the foreign body was precisely localized. The bladder mucosa and muscular layer were incised layer by layer with a circular electrode until the linear lesion was exposed in the muscular layer. The foreign body was then retrieved with the resectoscope (Figure 2F). Gross inspection confirmed a wooden toothpick (Figure 2G).

Postoperative abdominal ultrasound showed complete resolution of the linear hyperechoic lesion. Postoperative CT showed no residual high-density lesion in the bladder wall (Figure 2H). The patient received perioperative antibiotics, and she was discharged on postoperative day 1 with a urinary catheter retained for 1 week. No postoperative complications, including hematuria, occurred. At the one-week visit, the catheter was removed successfully. At six-month telephone follow-up, the patient reported marked improvement of abdominal and urinary symptoms and no new complaints, and she did not return for further testing.

## 3 Discussion

This case describes the diagnosis and treatment of a 29-year-old female patient admitted with lower abdominal pain and dysuria lasting one month. Initially, the patient's symptoms were mild and raised no significant concern. Medical attention was sought only when symptoms persisted and worsened. Accidental ingestion of foreign objects is common and typically requires no special intervention, as most foreign bodies are expelled spontaneously within a week (2). However, wooden toothpicks are difficult to digest due to their hard and sharp nature, often causing gastrointestinal perforation during passage through the duodenum or sigmoid colon, with an admission rate of approximately 12% following toothpick ingestion (14).

The most likely pathway for the wooden toothpick to enter the bladder in this case is as follows: During the patient's cesarean section, the bladder's peritoneal fold was incised, and the bladder was pushed downward. Due to prolonged postoperative healing, adhesions formed between the bladder and adjacent structures, such as the sigmoid colon, bringing them into closer proximity (15–17). After accidental ingestion, the toothpick perforated the sigmoid colon, migrated into the pelvis, and subsequently penetrated the bladder wall.

This case is particularly unique because the patient originated from Renshou County, Sichuan Province, China, where consuming wooden-toothpick beef is common, increasing the likelihood of accidental ingestion. Thorough interviews with the patient and family members revealed no history of mental disorders, and the patient had a healthy lifestyle. Considering dietary habits and relevant literature (7–11), we hypothesize that accidental ingestion and migration of the toothpick via gastrointestinal perforation occurred.

Typical symptoms of bladder foreign bodies include difficulty urinating (with or without urinary stream narrowing), gross hematuria (intermittent or persistent), bladder irritative symptoms (frequency, urgency, dysuria), suprapubic (lower abdominal) pain, and acute urinary retention (18, 19). Clinical symptoms vary significantly depending on the type of foreign body, its retention time, and infection severity (20, 21). Accidental ingestion of wooden toothpicks leading to gastrointestinal perforation and subsequent migration into the bladder is exceedingly rare. A PubMed search revealed only five documented cases, and their clinical features are summarized in Table 1. In this patient, the primary symptoms were lower abdominal pain and dysuria, consistent with typical presentations. The absence of significant peritonitis or abdominal infection could be attributed to two factors. First, the sharp toothpick slowly penetrated the intestinal wall and migrated into the bladder's muscular layer. The slow progression, driven by intestinal peristalsis, facilitated fibrous tissue formation, effectively isolating bacteria and intestinal contents. Second, the patient's immune response was adequate to control infection.

**Table 1:** Clinical characteristics of previously reported cases compared with the present case.

No.	Age	Gender	Reference	Clinical manifestations	Physical examination	Auxiliary examination	Treatment strategy	Postoperative recovery
1	54	Male	Di Migueli (7)	Symptoms of urinary tract infection (frequency, urgency, dysuria), abdominal colic	No significant abnormalities	Abdominal ultrasound: hyperechoic lesion in bladder wall; Cystoscopy: brown tubular structure with surface calcification, surrounding edema and congestion	Transurethral cystoscopic removal of bladder foreign body	Complete resolution of urinary and abdominal symptoms
2	23	Male	Tombolini, F (9)	Pneumaturia, fecaluria, and mild right lower abdominal pain	Deep palpation revealed tenderness in the right lower abdomen	Abdominal ultrasound: hyperechoic lesion in bladder wall; Cystoscopy: inflammatory lesion visible; Pelvic CT: foreign body in bladder wall identified	Failed cystoscopic removal, foreign body removed through laparoscopic cystotomy	Complete resolution of urinary and abdominal symptoms
3	31	Male	O'Dea, M. J (12)	Occasional dysuria	No significant abnormalities	Intravenous pyelography: inverted teardrop-shaped bladder; Cystoscopy: wooden toothpick penetrating the bladder wall	Transurethral cystoscopic removal of bladder foreign body	Complete resolution of urinary and abdominal symptoms
4	11	Male	Alagiri, M (11)	Abdominal pain, hematuria	No significant abnormalities	Abdominal ultrasound: hyperechoic lesion in bladder wall	Transurethral cystoscopic removal of bladder foreign body	Complete resolution of urinary and abdominal symptoms
5	78	Male	Garcia-Segui, A (10)	General malaise, high fever, frequency and urgency urinary incontinence	No significant abnormalities	Abdominal ultrasound: hyperechoic lesion in bladder wall; Pelvic CT: foreign body in bladder wall identified	Transurethral cystoscopic removal of bladder foreign body	Complete resolution of urinary and abdominal symptoms
6	29	Female	Present case	Persistent lower abdominal pain and frequent urination	No significant abnormalities	Abdominal ultrasound: hyperechoic lesion in bladder wall; Pelvic CT: foreign body in bladder wall identified	Transurethral cystoscopic removal of bladder foreign body	Complete resolution of urinary and abdominal symptoms

Diagnosing bladder foreign bodies requires a detailed medical history, comprehensive physical examination, and imaging studies. A literature review of 57 cases involving toothpick ingestion found 12% admitted swallowing the toothpick, 21% denied ingestion despite toothpick use, and 46% had risk factors for accidental ingestion (22). Symptom onset in admitted cases ranged from 1 day to 15 years, with symptom duration from 1 day to 9 months. Common risk factors include intellectual disability, rapid eating, alcohol abuse, and habitual chewing of toothpicks (23–25). This patient exhibited risk factors consistent with the literature, showing symptoms persisting for one month.

On physical examination, abdominal palpation or bimanual examination may detect large foreign bodies in thin patients. Abdominal x-rays effectively detect metallic objects but may fail to visualize non-metallic objects such as toothpicks. In such cases, abdominal ultrasound, a rapid and non-invasive tool, is preferred. Abdominal CT, due to its high resolution and multi-sequence imaging, has become essential for localization and diagnosis. It also distinguishes other causes of abdominal pain and clarifies anatomical relationships. In this case, CT imaging of a crucian carp with embedded needles allowed radiodensity comparison, ruling out a retained needle or fishbone. Based on dietary habits, a wooden toothpick was suspected. CT and ultrasound imaging precisely localized the foreign body in the posterior bladder wall. Transurethral surgery rather than laparoscopic or open surgery was selected for several reasons. Ultrasound-guided transurethral surgery permits precise localization and safer removal. Preoperative imaging indicated easier accessibility via the bladder lumen. In contrast, laparoscopic or open surgery involves more complex anatomical manipulation and greater tissue damage risks.

This study has several limitations. First, due to the rarity of this type of case, the diagnostic and therapeutic protocol was applied only to a single patient at a single center. Despite the successful clinical outcome, more cases and longer follow-up are necessary to fully assess this approach. Second, the migration path of the foreign body was not histologically confirmed.

## 4 Conclusion

This case highlights risks associated with wooden toothpick ingestion. The patient's dietary habits led to accidental swallowing and migration of a toothpick into the bladder, causing abdominal pain and dysuria. This emphasizes the importance of preventive education, especially in regions where toothpicks are commonly used in food preparation. Increasing public awareness about ingestion dangers through health education can help prevent such incidents. Clinicians should maintain vigilance when evaluating unexplained abdominal or urinary symptoms, considering possible foreign body ingestion to ensure timely diagnosis and appropriate treatment.

## Data Availability

The original contributions presented in the study are included in the article/Supplementary Material, further inquiries can be directed to the corresponding author.
